# Cure of Tinea Versicolor

**Published:** 1909-08

**Authors:** 


### CURE OF TINEA VERSICOLOR.

F. S. Mason, M. D., says this very annoying parasitical disease can be easily treated without inconvenience to the patient, by annointing the part effected with a one per cent. solution of mercuric iodide in oil (cypridol). This will immediately kill the parasite and within forty-eight hours all lesions will have disappeared. I have had no experience with its application to other skin diseases, but in tinea versicolor its effects are remarkably prompt and it is worthy of trial in ring worm and allied skin affections.

